# Practical Fast-Response Anodized-Aluminum Pressure-Sensitive Paint Using Chemical Adsorption Luminophore as Optical Unsteady Pressure Sensor

**DOI:** 10.3390/s22176401

**Published:** 2022-08-25

**Authors:** Yoshinori Oka, Takayuki Nagata, Miku Kasai, Yuta Ozawa, Keisuke Asai, Taku Nonomura

**Affiliations:** Department of Aerospace Engineering, Graduate School of Engineering, Tohoku University, 6-6-01 Aramakiaza-Aoba, Aoba-ku, Sendai 980-8579, Japan

**Keywords:** pressure-sensitive paint, anodized aluminum, H_2_TCPP, fast responsiveness, chemical adsorption

## Abstract

We developed and evaluated an anodized-aluminum pressure-sensitive paint (AA-PSP) with new formulations of free-base porphyrin, $H_{2}\mathrm{TCPP}$, as an optical unsteady pressure sensor. The luminophore $H_{2}\mathrm{TCPP}$ has quite a short fluorescent lifetime (2.4 ns on the condition of the AA-PSP). The fluorescence spectroscopy result shows that the excitation wavelength of $H_{2}\mathrm{TCPP}$ corresponds to violet-colored (425 nm) and green-colored (longer than 520 nm) lights. The pressure sensitivity is sufficiently high for the pressure sensor (0.33–0.51%/kPa) and the temperature sensitivity is very low (0.07–1.46%/K). The photodegradation of the AA-PSPs is not severe in both excitation light sources of the green LED and the Nd:YAG laser. The resonance tube experiment result shows the cut-off frequency of the AA-PSPs is over 9.0 kHz, and the results of the shock tube experiment show the 10 µs order time constant of the normal shock wave.

## 1. Introductions

A pressure-sensitive paint (PSP) [1] is a functional molecular sensor that enables contactless pressure measurement. It allows us to estimate the pressure distribution by measuring the fluorescence/phosphorescence intensity of the luminophore. The technique is applied to various fields such as fluid dynamics, aerodynamics, and acoustic investigations owing to its high spatial resolution. In aerospace engineering, a transonic buffet on a rocket-fairing [2,3,4] and a transport-sweep wing [5,6,7] are examples of typical applications. Measurements of pressure distribution in high spatial resolution allow us to investigate flow mechanics in detail. In addition, this technique is effective for models where it is difficult to install conventional pressure taps and models which where it is difficult to install pressure sensors, such as very thin wings [8,9,10], rotating wing [11,12,13,14,15,16], and free flight models [17].

A fast-responding PSP (fast PSP) is a relatively newly developed category of PSPs, enabling measurements with high spatial and temporal resolution. A variety of fast PSPs with response times of several hundred microseconds or fewer have been developed, which could provide high-frequency sampling measurements at the kilohertz order [18,19,20]. The time-resolved pressure fields yielded by a fast PSP have afforded valuable insight into complex flow phenomena from vortex-induced noise/vibration at low-speed flows [21] to shock–boundary-layer interactions at hypersonic flows [22]. A recent review article by Peng et al. [20] has focused on fast PSPs. A fast PSP is required to capture high-frequency pressure fluctuations under supersonic and transonic conditions. The time response of a PSP is limited by the diffusion coefficient of the binder and the luminescence lifetime of the luminophore. The luminescence lifetime is the duration of the luminescence caused by the transition of the dye molecules from the excited state to the ground state. The luminescence lifetime is the ultimate limit of the responding time of a PSP [23]. The gas diffusion phenomenon in a PSP binder is also considered to be a rate-determining factor in the responding time of a PSP. On the other hand, when a binder with sufficiently high gas diffusivity, the luminescence lifetime is non-negligible with respect to the time scale of gas diffusion in a PSP binder [24].

Many studies have developed polymer-ceramic PSPs (PC-PSPs) as fast PSPs [25,26,27]. Here, a PC-PSP is a mixture of a high concentration of ceramic particles with a small amount of polymer to physically hold the ceramic particles to a surface, where the ceramic particles bind with luminophore molecules. The PC-PSPs can achieve a kilohertz order cut-off frequency, but the surface roughness of them is not small. In general, the high-frequency phenomena are caused by high-speed flowfields, and they are affected by the surface roughness of the paint. Sugioka et al. [6,28] applied a PC-PSP with a low arithmetic surface roughness of 0.5 µm and a cut-off frequency of 3 kHz to transonic wind tunnel tests. This low surface roughness was achieved by polishing the paint surface. Peng et al. [29] developed a PSP with mesoporous silica, and the response time to a step pressure input was 100 μs. Egami et al. [30] improved the response time of a sprayable PC-PSP using tris(bathophenanthroline) ruthenium dichloride (Ru(dpp)${}_{3}$) to a microsecond order. Kasai et al. [24] reported the diffusion coefficient of a PC-PSP is relatively high as a fast PSP. However, the improvement of responding capability leads to a reduction in paint durability. Peng et al. [31] reported that coatings with near-surface luminophore distribution are quite fragile to mechanical damage in high-speed applications. Reductions in thickness and polymer concentrations to improve response would reduce the mechanical strength of the paint.

Anodized aluminum-PSPs (AA-PSPs) have also been developed as one of the fast PSPs. AA-PSPs use the porous structure of the anodized aluminum layer as the binder of the luminophore. Asai et al. [32] and Sakaue et al. [33] proposed the AA-PSP and used Ru(dpp)${}_{3}$ as a luminophore. An anodized aluminum layer is fabricated by an anodization process of an aluminum model. The anodization process is well established and highly repeatable [34]. An AA-PSP is fabricated by adsorbing a luminophore on the anodized aluminum layer of the model. There are two adsorption types: physical adsorption and chemical adsorption. The durability of an AA-PSP is quite higher than other PSPs. The luminophore of an AA-PSP is exposed to the atmosphere and it has a relatively high diffusivity coefficient of the porous surface as a binder [24]. AA-PSPs have been employed for pressure measurements in various flowfields [22,35,36]. Egami et al. [37] and Kameda et al. [23] investigated the characteristics of luminophores, such as pyrene, ruthenium complexes, and porphyrins. A fast AA-PSP using pyrene as a luminophore is widely researched. Pyrene has a quite short luminescence lifetime $O\left(10\right)$ ns [23]. Numata et al. [38] developed an ultra-fast AA-PSP, which uses 1-Pyrene butyric acid (PBA) as a luminophore. They anodized aluminum using phosphoric acid as an electrolyte. The rise time to reach 90% in the pressure signal of their AA-PSP is 0.81 μs. Yomo et al. [39] have used pyrene sulfonic acid (PSA) as a luminophore. They investigated the effects of the kind of solvent, luminophore concentration, and anodizing time. Pyrene has good characteristics in time response and pressure sensitivity. However, pyrene has a severe photodegradation characteristic and the evaporation of the pyrene under wind tunnel blow down conditions [40]. Although it is not a serious problem when the measurement time is very short, such as measurement using a ballistic range, this characteristic makes it difficult to obtain practical measurement time in a typical wind tunnel experiment.

The free-base porphyrin compound tetrakis(4-carboxyphenyl) porphyrin (TCPP) is a prospective luminophore for a fast PSP because of its luminescence lifetime (3.2 ns) [23]. The luminescence lifetime of a TCPP is shorter than that of pyrene. This characteristic corresponds to the fact that a TCPP is faster responding than pyrene, so it is potentially a faster AA-PSP.

Amao and Okura [41] investigated the characteristics of AA-PSP using a TCPP with and without metal complex as a luminophore. They reported that free-base porphyrin ($H_{2}\mathrm{TCPP}$) showed good response time but the oxygen sensitivity was lower than TCPPs with metal complexes. Takeuchi and Amao [42] reported that a TCPP is chemically adsorbed on the anodized aluminum layer, and it may possess a lower diffusion barrier for oxygen. The other advantage of chemical adsorption is the strength of bonding to the anodized aluminum layer. The chemical adsorbed luminophore cannot be removed with any polar solvents [23]. In the present study, we focused on an $H_{2}\mathrm{TCPP}$. It has a quite short luminescence lifetime and very low-temperature sensitivity [43] and photodegradation rate. However, it does not have sufficiently high signal intensity for the single-shot PSP measurement. In the present study, the solvent, luminophore concentration, and pore configuration of the binder have been changed from the previous research, and the effect of that on the performance of the pressure sensor has been investigated. Moreover, the time-series images of the pressure distribution caused by the normal shock wave propagation were acquired by a high-speed camera.

## 2. Pressure-Sensitive Paint

### 2.1. Basic Principles of PSP

The basic principles of a PSP have been detailed by Liu et al. [1]. A PSP comprises a luminophore, an oxygen-sensitive luminescent molecule, and a binder material to hold the luminophore on the model surface. The luminophore is excited to a heightened energy state by the adsorption of photons from an excitation light source. The energy of the excited state can be released through various mechanisms such as Stokes-shifted luminescence at a longer wavelength, oxygen quenching, and thermal deactivation. The oxygen quenching of the luminescence is the fundamental principle of a PSP. A higher oxygen concentration increases quenching and reduces the intensity of luminescence of the PSP. The luminescent intensity of the paint is a function of the partial pressure of oxygen in the air. There are two primary methods for acquiring the pressure distribution from a PSP: intensity-based and lifetime methods. In the present study, the intensity-based method was applied. The intensity-based method usually uses continuous excitation light and measures the signal intensity of luminescence from the PSP. The raw luminescence signal intensity cannot be directly expressed by the pressure distribution because the measured signal intensity contains the dependency of the ambiguity resulting from the spatially inhomogeneous excitation light intensity, the paint thickness, the luminophore concentration, etc. Reference images acquired at known pressure conditions are required for the calculation of the signal intensity ratio of the two images. The signal intensity ratio is related to pressure by the Stern–Volmer-type relationship, which is normalized by the reference condition and Equation (2) [1], as follows:

(1) $$\frac{I_{\mathrm{ref}}}{I}=A_{1}\times \frac{P}{P_{\mathrm{ref}}}+A_{2},$$

(2) $$\frac{I}{I_{\mathrm{ref}}}=B_{1}T^{2}+B_{2}T+B_{3},$$

where $I_{\mathrm{ref}}$ and *I* are the signal intensity images at the reference (wind-off) and test (wind-on) conditions, *A* and *B* are pressure- and temperature-dependent calibration coefficients, and *P* and $P_{\mathrm{ref}}$ are the pressure distributions corresponding to the wind-on and wind-off conditions, respectively. Instrumentation for the intensity-based method is generally composed of a light-emitting diode (LED) excitation light source, with a charge-coupled device (CCD) or complementary metal oxide semiconductor (CMOS) camera as a photodetector.

### 2.2. Anodized-Aluminum Pressure-Sensitive Paint Characteristics

The frequency response of a PSP is generally lower than that of pressure transducers, and much research on fast PSPs has been conducted towards the application to the measurements of high-frequency unsteady phenomena. An AA-PSP [32,44] is one of the fast PSPs. The responsiveness of an AA-PSP is dominated either by the diffusion time of the oxygen or by the luminescence lifetime of the luminophore. Winslow et al. [45] showed that the time to reach 99% total variation of luminescence of a PSP can be estimated by Equation (3).

(3) $$t_{0.99}=1.8\frac{h^{2}}{D}$$

where $t_{0.99}$ is the characteristic time, *h* is the thickness of the anodized aluminum layer, and *D* is the diffusion coefficient. The units of $t_{0.99}$, *h*, and *D* are s, m, and m${}^{2}$/s, respectively. The diffusion coefficient is expressed by the Knudsen diffusion and bulk diffusion coefficients, as in Equation (4).

(4) $$D=\frac{D_{kg}D_{gg}}{D_{kg}+D_{gg}},$$

where $D_{kg}$ and $D_{gg}$ denote the diffusion coefficients of Knudsen diffusion and bulk diffusion, respectively. The gaseous transport is dominated by collisions with the pore walls when the mean free path of gas molecules exceeds the pore diameter. This process is known as Knudsen diffusion [23]. The coefficient of Knudsen diffusion is

(5) $$D_{kg}=\frac{d}{3}\sqrt{\frac{8RT}{\pi M}},$$

where *d*, *R*, and *M* are the pore diameter, the universal gas constant, and the molecular weight of the gas, respectively. Equation (5) represents that the Knudsen diffusion is proportional to the pore diameter. Therefore, an increase in the diffusion coefficient by increasing the pore diameter and by decreasing the coating thickness can improve the response.

The signal intensity of luminescence is also related to the pore diameter and the thickness of the anodized aluminum layer. In general, the larger pore diameter and the thinner anodized aluminum layer decrease the emission intensity of the PSP in contrast to the increase in the response of the PSP [19,38]. The time response of the PSP is limited by the diffusion coefficient of the binder and the luminescence lifetime of the luminophore. The diffusion coefficient is limited by the shape of the pore structure, which means there is a limitation to improving the diffusion coefficient. It is effective to choose a luminophore with a short luminescence lifetime for improvement of the response of the PSP. Pyrene is a prospective luminophore for a fast PSP because it has a quite short luminescence lifetime of $O\left(10\right)$ ns [23]. Pyrene also has high luminescence intensity and this characteristic is very attractive to apply for high sampling rate measurement because the exposure time of the photodetector could be minimized. However, pyrene has a severe photodegradation characteristic and this characteristic makes it difficult to obtain practical measurement time in a typical wind tunnel experiment. Egami et al. [37] and Kameda et al. [23] used a free-base porphyrin on the AA-PSP, and they reported that it has high responsiveness and resistance to photodegradation but the signal intensity is not sufficiently high for an application to pressure measurements. They also reported that the response time is independent of the thickness of the anodized aluminum layer. Therefore, in the present study, the new AA-PSP was developed using a free-base porphyrin as a luminophore with a different solvent, luminophore concentration, and excitation light source from previous studies for the application of pressure distribution measurement of supersonic flow fields.

## 3. Experimental Apparatus

The eight samples were fabricated with different preparation conditions. The reference sample was fabricated with dilute sulfuric acid as the electrolyte and anodized for 20 min. The preparation conditions of fabrication were kinds of electrolyte, anodization time, and luminophore concentration. The sample-based parameter study was conducted, and the characteristics of each sample, such as pressure sensitivity, temperature sensitivity, photodegradation rate, signal intensity, and frequency response, were obtained. The excitation and emission wavelength and luminescence lifetime were investigated on the reference sample. The static calibration and photodegradation tests were performed on all the samples. The responsiveness of the samples which have good static characteristics was evaluated via dynamic calibration tests, such as acoustic resonance tube and shock tube experiment.

### 3.1. Materials and Luminophore Solution

The anodized aluminum layer of the AA-PSP is fabricated by an anodizing process of the samples. The thicknesses of the anodized aluminum layer and the pore diameter are related to the anodizing time and the kinds of the electrolyte, respectively. In the present study, a standard AA-PSP fabrication process is adopted:

(1) Pre-treatment
  Pure aluminum samples were soaked into 3% sodium hydroxide solution a few minutes. Pure aluminum plates were rinsed with distilled water after the soaking process. Then, the plates were dried in vacuum desiccators for several hours.
(2) Anodization
  Two types of electrolytes, sulfuric acid and phosphoric acid, were used in the anodizing process. The post-treatment process is different for the electrolyte. The samples were anodized with a constant current density of 12.5 mA/cm${}^{2}$. The sample was connected to the anode in 1 molar sulfuric acid in 10 ${}^{\circ }C$ or 1 molar phosphoric acid in 30 ${}^{\circ }C$. After the anodization process, the samples were rinsed with distilled water and dried in vacuum desiccators for several hours in a vacuum desiccator.
(3) Post-treatment
  The anodized samples were soaked into 3% phosphoric acid for 20 min at a constant temperature (20–30 ${}^{\circ }C$) in the case of fabrication by using the sulfuric acid electrolyte or 60 min at a constant temperature (20–30 ${}^{\circ }C$) in the case of fabrication by using the phosphoric acid electrolyte. Then, the samples were then rinsed with distilled water and dried in vacuum desiccators for several hours.
(4) Luminophore adsorption
  The sample is dipped into the luminophore ($H_{2}\mathrm{TCPP}$) solution for 100 s. Then, the sample is quickly rinsed with pure acetone and the inhomogeneous adsorption of luminophore is reduced. Finally, it is dried at least overnight in a vacuum desiccator.

In the present study, eight types of AA-PSPs samples were fabricated. Their fabrication conditions and characteristics are summarized in Table 1. Phosphoric acid and dilute sulfuric acid were used as electrolytes for the anodization process and the effects of the pore diameter on pressure sensitivity, signal intensity, and responsiveness were investigated, respectively. The size of the samples for static characteristics investigation was 15 × 20 mm. Samples were fabricated by varying the anodization time from 10 to 30 min and 20 to 60 min in the case of dilute sulfuric acid anodization and phosphoric acid anodization, respectively, and the effects of the thickness of the anodized aluminum layer, which depends on the anodization time, on static characteristics and responsiveness were investigated. The thickness of the anodized aluminum layer is proportional to the anodizing time, as in a previous study [46]. The thickness of the anodized aluminum layer was measured using an eddy current film thickness meter (LZ-373, Kett, Tokyo, Japan) with a measurement precision of ±1 µm. In the case of Ru(dpp)${}_{3}$ and PBA, the signal intensity decreases and the responsiveness increases as the thickness of the anodized aluminum layer becomes thinner. On the other hand, in the case of $H_{2}\mathrm{TCPP}$, the thickness of the anodization aluminum layer does not impact the responsiveness, as shown in a previous study [23]. Samples of 0.01 mM and 0.9 mM were fabricated and the effects of the luminophore concentration on static characteristics were investigated. The thickness of these two samples is the same as that of the reference plate. The luminophore concentration is 0.1 mM, except for these two samples. The solvent resistance of the samples was investigated. The samples after the laser photodegradation test were used in the solvent resistance investigation. The effect of the acetone rinsing on the pressure and temperature sensitivities was investigated by static calibration.

Ethanol, methanol, and dichloromethane were used as solvents for $H_{2}\mathrm{TCPP}$ in the previous research [23]. In these cases, sufficiently high signal intensities were obtained with an exposure time of the order of milliseconds. In the present study, pure acetone was employed as a solvent, as it is expected to provide the highest signal intensity according to the previous comparative study on the AA-PSP characteristics conducted by Sakaue et al. [47]. The standard AA-PSP, ${\mathrm{SA}}_{\mathrm{Ru}}$, was also fabricated for the purpose of the signal intensity comparison with the proposed AA-PSP. The fabrication conditions of the anodized aluminum layer were the same as the reference sample. The luminophore for the standard AA-PSP was Ru(dpp)${}_{3}$. We prepared a luminophore solution consisting of 11.7 mg of the luminophore dissolved in 100 ML of the solvent, which was dichloromethane [47]. The duration of dipping for luminophore was 10 s. Spectroscopic and lifetime measurements were performed on the reference plate.

### 3.2. Fluorescence Spectroscopy

**Figure 1:**
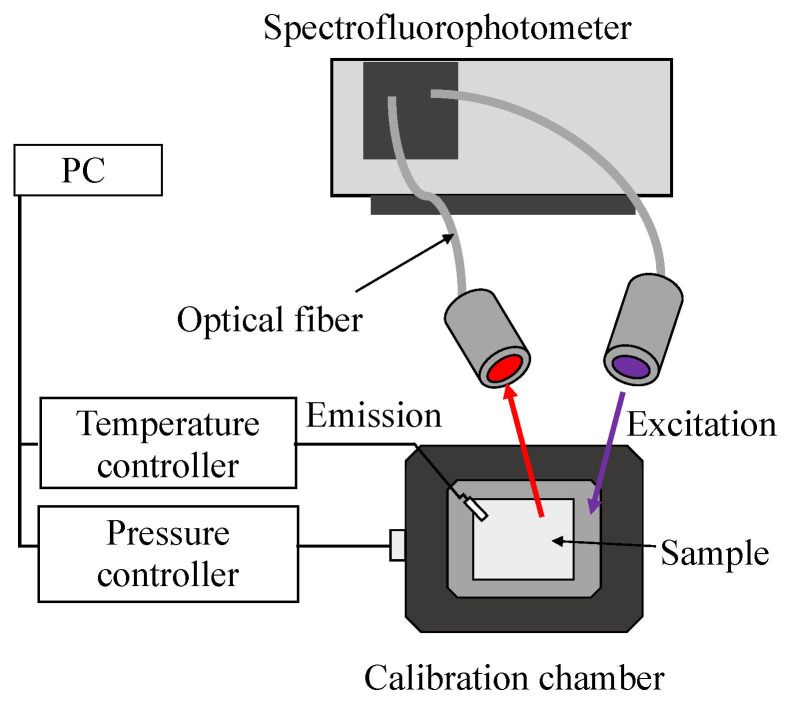
Experimental setup of fluorescence spectroscopy.

The excitation spectrum of the reference plate and emission spectrum of the reference plate and ${\mathrm{PA}}_{T60}$ were investigated by fluorescence spectroscopy (RF-5300C, Shimazu, Kyoto, Japan), as schematically shown in Figure 1. The reference plate was placed inside a chamber in which the pressure and the temperature are controllable. The pressure inside the chamber *P* and the temperature of the reference plate *T* were 100 kPa and 293 K, respectively. The pressure dependency of the emission wavelength of the reference plate was also investigated, whereas the excitation wavelength was fixed at 532 nm, and the pressure inside the chamber *P* and temperature of the reference plate were 10–140 kPa and 293 K, respectively. These investigations were performed with 1 nm increments of the wavelength. A 440 nm long pass filter was installed between the photodetector and the reference plate.

### 3.3. Static Calibration Chamber

**Figure 2:**
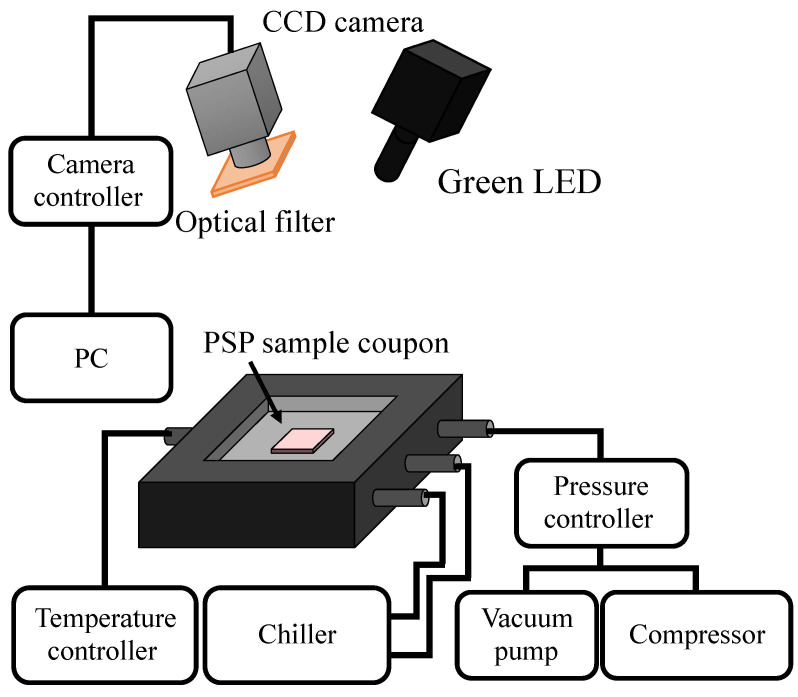
Experimental setup of the chamber.

The pressure and temperature sensitivities of samples were obtained using a static calibration chamber, as schematically shown in Figure 2. The samples were placed in a calibration chamber in which the pressure and the temperature are controllable. The pressure inside the chamber *P* and the temperature of the samples were 10–120 kPa and 278–303 K, respectively. A green LED (IL-106, HARDsoft, Krakow, Poland) with a central wavelength of 528 nm was used as the excitation light source. A 540 nm short-pass filter was installed between a green LED and the samples. The power output of the LED excitation source was 10 W. The emissions from the samples were detected by a 16-bit CCD camera (C4742-98, Hamamatsu Photonics, Shizuoka, Japan). A camera lens with a focal length of 105 mm (Nikkor 105 mm f2.8, Nikon, Tokyo, Japan) was attached to the CCD camera with a 640±50 nm band-pass filter (PB0640-100, Asahi, Tokyo, Japan). The pressure sensitivity, temperature sensitivity, and photodegradation analyses were performed on an image of each sample, and the standard deviation in the spatial direction was used to evaluate the uncertainty of each quantity. The effect of the acetone rinsing process on the pressure and temperature sensitivities was investigated in the same manner.

### 3.4. Laser and Camera for Laser Photodegradation

**Figure 3:**
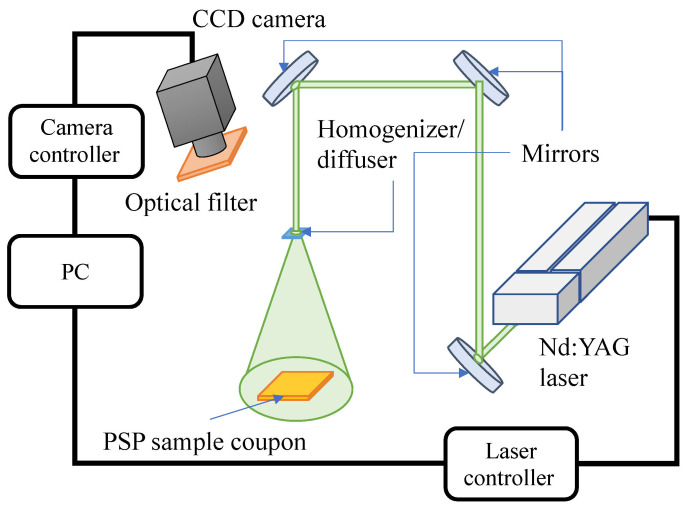
Experimental setup of laser photodegradation.

The laser photodegradation rates of the samples were obtained from the signal intensity of luminescence when excited by a pulsed Nd:YAG laser. The experimental setup is shown in Figure 3. The pulsed Nd:YAG laser with a central wavelength of 532 nm was used as the excitation light source. The output energy of the Nd:YAG laser was approximately 2 mJ/pulse. The laser beam was converted to a uniform round shape by a homogenizer/diffuser (#14-683, Edmund, Barrington, NJ, USA) and illuminated the samples. The distance between the samples and homogenizer/diffuser was 1.7 m, and the diameter of the diffused laser beam at the plane of the samples was approximately 190 mm. The emission from the samples was detected by a 12-bit high-speed camera (SA-X2, Photoron, Tokyo, Japan). The camera lens (Nikkor 50 mm f1.2, Nikon, Tokyo, Japan) with a focal length of 50 mm was attached to the camera with a 640±50 nm band-pass filter (PB0640-100, Asahi, Tokyo, Japan). This measurement was conducted under atmospheric conditions.

### 3.5. Picosecond Laser and Streak Camera

In the luminescence lifetime measurement, the reference plate was excited using a diode-pumped picosecond Nd:YAG laser (PL2210, Hamamatsu, Shizuoka, Japan). The wavelength of the Nd:YAG laser was 532 nm. The energy output of excitation was 0.45 mJ/pulse. The width of the laser pulse was 28 ps. The emission of the reference plate was captured by a streak camera (C7700, Hamamatsu, Shizuoka, Japan). The measurement was conducted under atmospheric conditions. The obtained emission response curve was approximated by the double-exponential function shown in Equation (6):

(6) $$f\left(t\right)=\alpha \exp\left(-\beta t\right)+\gamma \exp\left(-\delta t\right),$$

The luminescence lifetime was defined as the duration from the time of maximum luminescence intensity to the time of 90% decay of the luminescence intensity. The luminescence lifetime was calculated from an approximate curve using the double exponential function shown in Equation (6).

### 3.6. Resonance Tube

**Figure 4:**
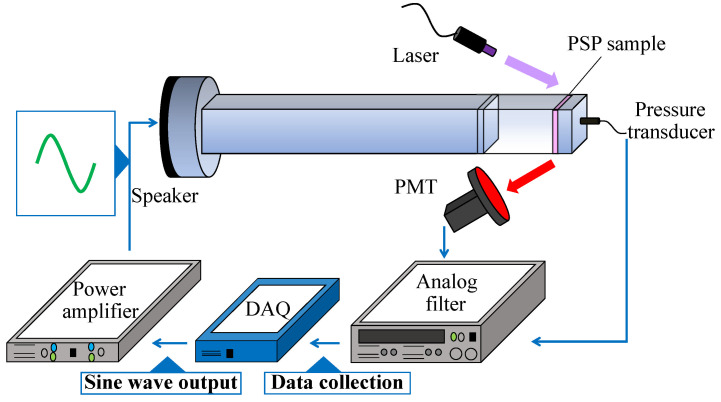
Schematic illustration of a resonance tube.

The dynamic characteristics of AA-PSPs were investigated by a frequency response test with an acoustic resonance tube [48], as shown in Figure 4. The speaker is installed at one of the ends of the acoustic resonance tube, and the sinusoidal pressure oscillations are generated on the order of kilopascals in the frequency range of 0.15–10 kHz. The other end of the tube was capped by a PSP sample with a hole at the center of it, and a pressure transducer (XCL-152-5SG, Kulite, Leonia, NJ, USA) was installed in the hole. The size of a PSP sample is 20 × 20 mm. The temperature measuring resistor (R060-39, Chino Corporation, Tokyo, Japan) and the Peltier device (FPH1-12706AC, Fujita Corporation, Tokyo, Japan) were installed on the back of the sample, and the temperature of the sample could be controlled by the Peltier controller (TD-1000A, Cell System Corporation, Kanagawa, Japan). The PSP was excited using the ultraviolet (UV) laser (RV-1000TH, Ricoh, Tokyo, Japan) with a wavelength of 400 nm. The distance between the sample and the laser was set to be approximately 400 mm. The emission from the sample was measured using the photomultiplier tube (PMT; H5784-02, Hamamatsu , Shizuoka, Japan). The 640±50 nm band-pass filter (PB0640-100, Asahi, Tokyo, Japan) was placed in front of the PMT. The pressure was measured at the same time as the PSP measurement with the pressure transducer installed in the center of the PSP sample. The signals obtained by the PMT and the pressure transducer were recorded simultaneously with the data acquisition (DAQ) device (USB-6251, National Instruments, Austin, TX, USA). The output part has the speaker. The high-frequency speaker (RX22, Peavey, Meridian, MS, USA) was employed and the frequency range of measurements was set to 0.5–10 kHz. The number of input cycles from the power amplifier (CP600, Classic Pro, Chiba, Japan) to the speaker was 4210 cycles. The amplitude of the output power from the power amplifier to the speaker was approximately 0.125 to 0.5 W, depending on the frequency. The pressure in the acoustic resonance tube was atmospheric pressure. The recorded signals of the PMT were then converted to pressure using an in situ calibration result of the PSP at the lowest frequency (0.5 kHz). The gain and phase delays of the PSP signal were calculated by comparing the amplitude and phase of the signals obtained by the pressure transducer. The cut-off frequency, which is the frequency at which the gain attenuation is −3 dB, was used as an index of the frequency response of the PSP.

The diffusivity coefficients *D* of the AA-PSPs were estimated from the obtained gain and phase delays by fitting the frequency response of the two-layer PSP model proposed by Nonomura and Asai [49]. The two-layer PSP model has been proposed, but it can be applied to single-layer PSPs, such as AA-PSPs. The hiding factor and the thickness of the second layer were approximated as zero. The harmonic pressure response was measured for $P_{\omega }$ and compared with a low-frequency approximation $P_{\omega ,\mathrm{ideal}}$, and $P_{\omega }/P_{\omega ,\mathrm{ideal}}$ was firstly calculated from the obtained gain and phase delay of the AA-PSP signal, and the frequency response data were approximated to the response model by changing the diffusivity in the model. The STD of $P_{\omega }/P_{\omega ,\mathrm{ideal}}$ was calculated from the standard deviation (STD) of the gain and phase delay for the same measurement. The approximation was performed with the gradient descent: each parameter was optimized to minimize the squared Frobenius norm of the difference between the experimental value and the model value of $P_{\omega }/P_{\omega ,\mathrm{ideal}}$. Here, the squared Frobenius norm was calculated after multiplying the difference between the model and the experimental value $P_{\omega }/P_{\omega ,\mathrm{ideal}}$ by the reciprocal of STD, which corresponds to the reliability of the data, as a weighting function. The initial diffusion coefficient of the optimization using gradient descent was $1.54\times 10^{-6}$ m${}^{2}$/s. The iterative calculation was stopped when the residual, which is the difference between the values of an objective function at a previous and a current step, was smaller than $10^{-10}$. The estimated cut-off frequency was calculated as that at which the gain of the model approximated by the frequency response would be −3 dB.

### 3.7. Shock Tube

**Figure 5:**
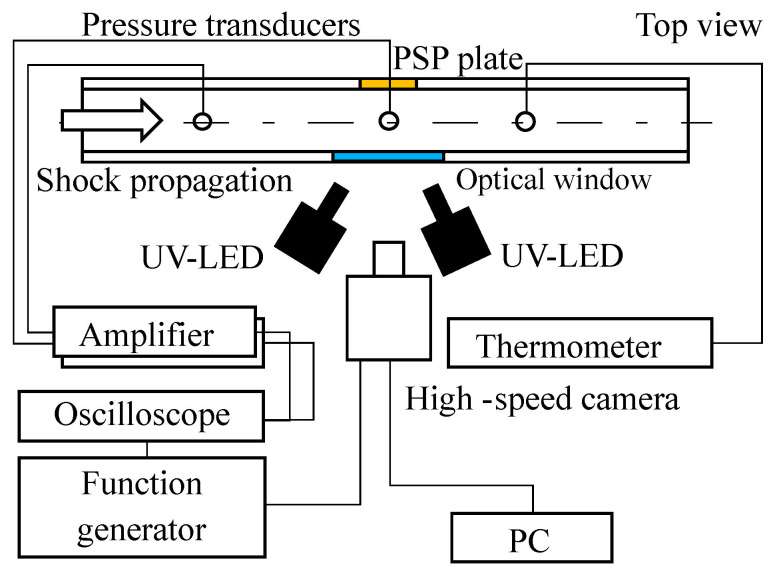
Scheme of the experimental setup for shock wave measurement.

Experiments for time response evaluation were performed in the diaphragmless 100×180 mm shock tube at the Institute of Fluid Science, Tohoku University. This shock tube has good repeatability and the variance of the shock Mach number is ±0.3% for the shock Mach number from 1.2 to 5.0 in air [50]. Figure 5 shows a schematic diagram of the shock tube experiment. The test gas and driver gas were dry air at room temperature. Two pressure transducers (Type 603B, Kistler, Winterthur, Switzerland) were installed on the upstream and the center of the region of interest, respectively. The signals from the pressure transducers were recorded by an oscilloscope (DSOX1204G, Keysight, Santa Rosa, CA, USA). These pressure transducers were used as the source for calculating the velocity of the shock wave and worked as a measurement trigger. The sample was installed in the side wall of the shock tube, and the diameter of the sample was 60 mm. In this experiment, two UV LEDs (IL-106, HARDsoft, Krakow Poland) with central wavelengths of 395 nm and 400 nm were used as the excitation light source. The radiometric flux of the LED excitation source was 16 W in total. The emissions from the samples were detected by a 12-bit high-speed camera (Phantom v2640, Vision Research, Wayne, NJ, USA). A camera lens (Nikkor 50 mm f1.2, Nikon, Tokyo, Japan) was attached to a camera with a 580 nm long-pass filter (O58, Hoya Optronics, Tokyo, Japan). The exposure time of the high-speed camera was 1.6 μs. The normal shock wave was visualized based on the intensity method. The wind-off and wind-on images were processed and the signal intensity ratio was obtained. The time-averaged wind-off image was used as a reference image, and it was an ensemble-averaged image of 1000 images before the arrival of the shock wave. The Wiener filter of 3 × 3 pixels was applied to the wind-on and wind-off images for noise reduction.

## 4. Results and Discussions

### 4.1. Static Characteristics

#### 4.1.1. Excitation and Emission Spectrum

**Figure 6:**
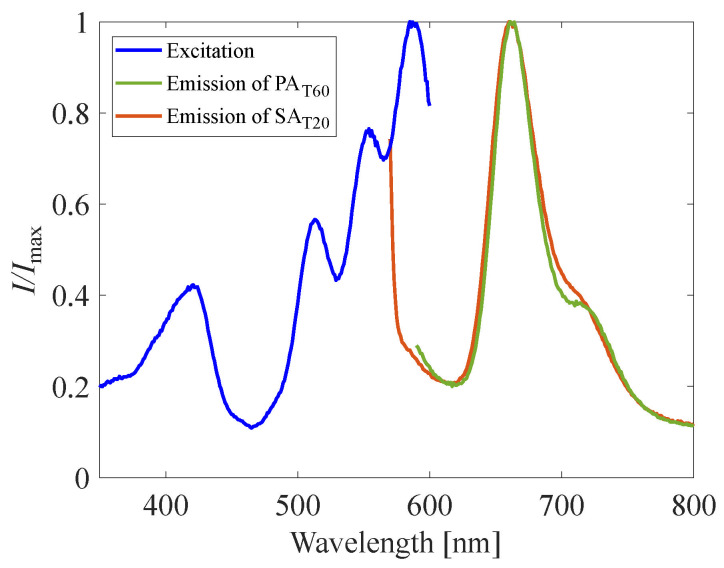
Excitation and emission spectrum of the reference plate.

**Figure 7:**
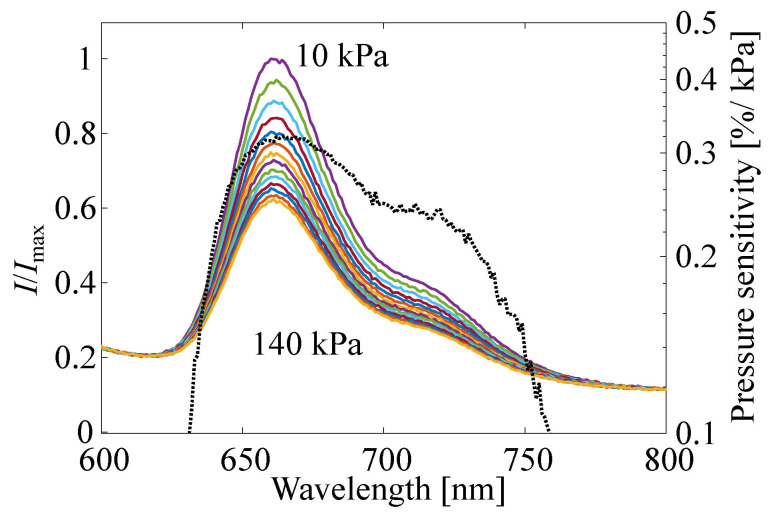
Emission spectrum at each pressure excited by 532 nm wavelength light.

Figure 6 shows the excitation spectrum of the reference plate and emission spectrum of the reference plate and ${\mathrm{PA}}_{T60}$. The excitation spectra of the reference plate show two high-intensity spectra, which are around 425 nm and also longer than 520 nm. The central emission wavelength of the reference plate and ${\mathrm{PA}}_{T60}$ are 660 nm and 661 nm, respectively. There are no significant differences in the stokes shift between the reference plate and ${\mathrm{PA}}_{T60}$, while there is a difference in the excitation spectrum around 700 nm. The emission intensity around 700 nm from ${\mathrm{PA}}_{T60}$ is weaker than that of the reference plate, and it can suggest the difference in the deactivation process, such as thermal quenching [51]. The excitation wavelength longer than 520 nm suggests that a Nd:YAG laser and a Nd:YLF laser are valid as excitation light sources. These lasers are widely used in PIV measurements and have the capability of high-power and high-repetition-frequency illumination. The combination of $H_{2}\mathrm{TCPP}$ and lasers has a high potential for high-frequency measurements. Figure 7 shows the emission spectra in solid lines and the pressure sensitivity of each wavelength in dashed lines. The emission spectra is obtained in the condition P=10–140 kPa, where the excitation wavelength was fixed at 532 nm for this measurement. The emission intensity clearly decreases as the pressure increases. The pressure sensitivity of each wavelength is calculated based on the signal intensity of the emission spectra. The peak emission wavelengths did not change from 660 nm at each pressure, and the wavelength where the pressure sensitivity becomes the maximum is 665 nm, which almost corresponds to the central emission wavelength. There are secondary profiles around 720 nm, but they show less pressure sensitivity than that around the peak emission wavelength.

#### 4.1.2. Luminescence Lifetime

**Figure 8:**
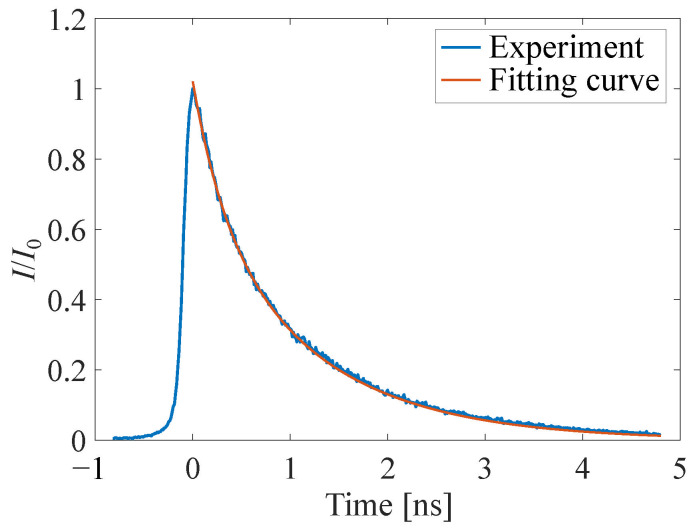
Luminescence lifetime of $H_{2}\mathrm{TCPP}$ on the reference at atmospheric conditions.

Figure 8 shows the result of the luminescence lifetime measurement of the reference plate. The blue solid line is the measured intensity decay and the red solid line is the approximate curve, as shown in Equation (6). The luminescence lifetime imposes an ultimate limit on the time response with which all the pressure-sensitive coatings can respond to pressure changes. Previous studies on the luminescence lifetime of $H_{2}\mathrm{TCPP}$ as an AA-PSP have shown that the luminescence lifetime is approximately 3.8 ns in air [23]. The luminescence lifetime of the reference plate at atmospheric conditions is 2.32 ns and is almost the same as that of the value reported in the previous study [23]. It should be noted that the luminescence lifetime measurement result is not affected by the fall time of the excitation light source because the width of the laser pulse was one-hundredth of the measured lifetime of $H_{2}\mathrm{TCPP}$. In the present study, the luminescence lifetime of the AA-PSP was confirmed to be sufficiently short compared to the diffusion.

#### 4.1.3. Signal Intensity

**Figure 9:**
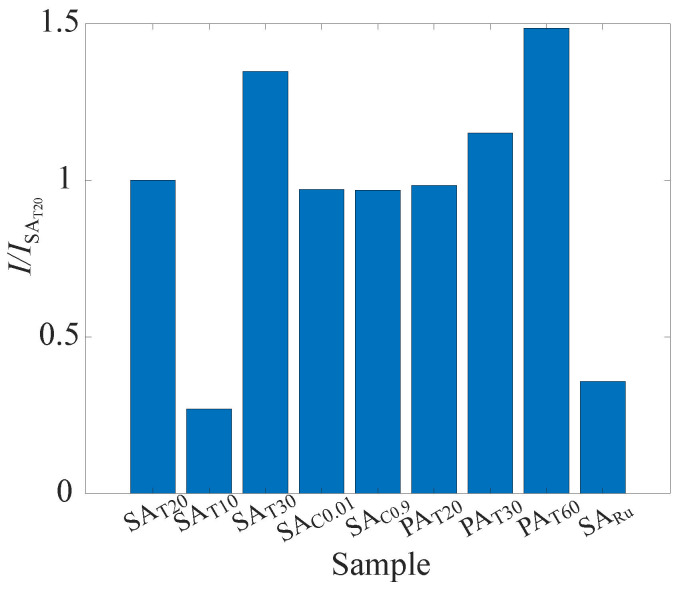
Influence of preparation conditions on the luminescence signal intensity.

Figure 9 shows the comparison of the signal intensity. A green LED was employed as the excitation light source. The signal intensity was acquired at a temperature of 293 K and pressure of 100 kPa. A dark current was subtracted from the signal intensity. The signal intensity of the samples is normalized by that of the reference plate. Here, ${\mathrm{PA}}_{T60}$ has the highest value of the signal intensity, followed by ${\mathrm{SA}}_{T30}$, ${\mathrm{PA}}_{T30}$, and ${\mathrm{SA}}_{T20}$. In both electrolyte cases, the signal intensity tends to increase as the thickness of the anodized aluminum layer increases. The signal intensity was generally higher in the case of the phosphoric acid electrolyte than in the case of the dilute sulfuric acid electrolyte in the present study. Note that the surfaces of the AA-PSP samples were tarnished after the anodizing process, and they became matte white in phosphoric acid electrolyte cases. Here, the value of the signal intensity of ${\mathrm{SA}}_{\mathrm{Ru}}$ is 64.2% lower than that of ${\mathrm{SA}}_{T20}$. The difference in the excitation spectra was considered to cause this result. The high absorption spectrum of Ru(dpp)${}_{3}$ is 455 nm, a blue color light, while the absorption spectrum around a green color light is very low [52]. As mentioned in the introduction, the low signal intensity of $H_{2}\mathrm{TCPP}$ was one of the challenges for PSP measurement. The comparison of the signal intensity between ${\mathrm{SA}}_{\mathrm{Ru}}$ and ${\mathrm{SA}}_{T20}$ reinforces that the PSP measurement system that combines the proposed AA-PSP and a laser, such as a Nd:YAG laser, could overcome the low signal intensity challenge.

#### 4.1.4. Pressure and Temperature Sensitivities

**Figure 10:**
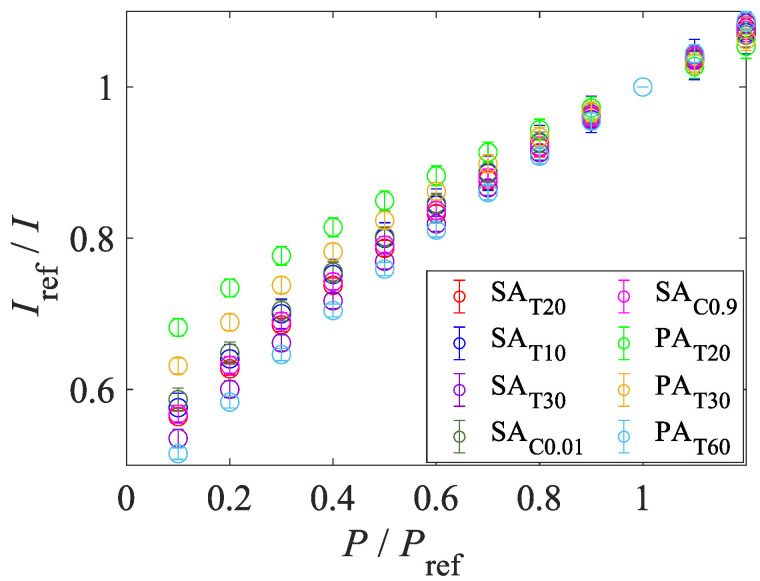
Influence of preparation conditions on Stern–Volmer-type plots.

**Figure 11:**
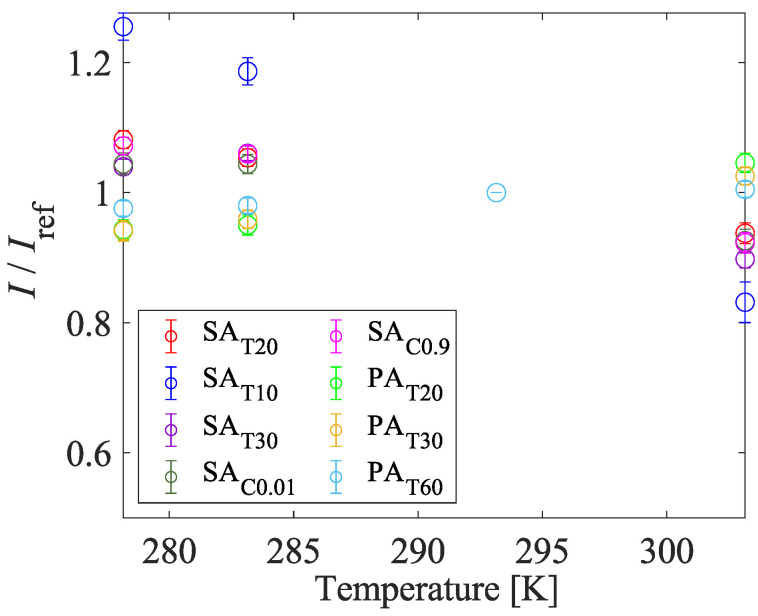
Influence of preparation conditions on temperature calibration curves.

Figure 10 shows the Stern–Volmer-type curves. The temperature of the sample was fixed at 293 K. The Stern–Volmer-type curves of all samples are not linear at low pressure. The gradient of the Stern–Volmer-type curve becomes steeper at low pressure. This trend indicates that the AA-PSPs show better pressure sensitivity at low pressure and it is suitable for the suction type supersonic wind tunnel experiment. Figure 11 shows the temperature calibration curves. The temperature sensitivity of the AA-PSP samples was evaluated at 100 kPa. The performance of a PSP was evaluated based on pressure sensitivity and temperature sensitivity. The pressure and temperature sensitivities, $S_{P}$ and $S_{T}$, were evaluated by the slope of Equation (1) and the absolute value of the slope of Equation (2) at each reference condition:

(7) $$S_{P}=\frac{\partial (I_{\mathrm{ref}}/I)}{\partial (P/P_{\mathrm{ref}})}÷\frac{P_{\mathrm{ref}}}{100}=\frac{100A_{1}}{P_{\mathrm{ref}}}\left[\%/\mathrm{kPa}\right],$$

(8) $$S_{T}=\left|\frac{\partial (I/I_{\mathrm{ref}})}{\partial T}\right|\times 100=\left|2B_{1}T_{\mathrm{ref}}+B_{2}\right|\times 100\left[\%/K\right].$$

**Figure 12:**
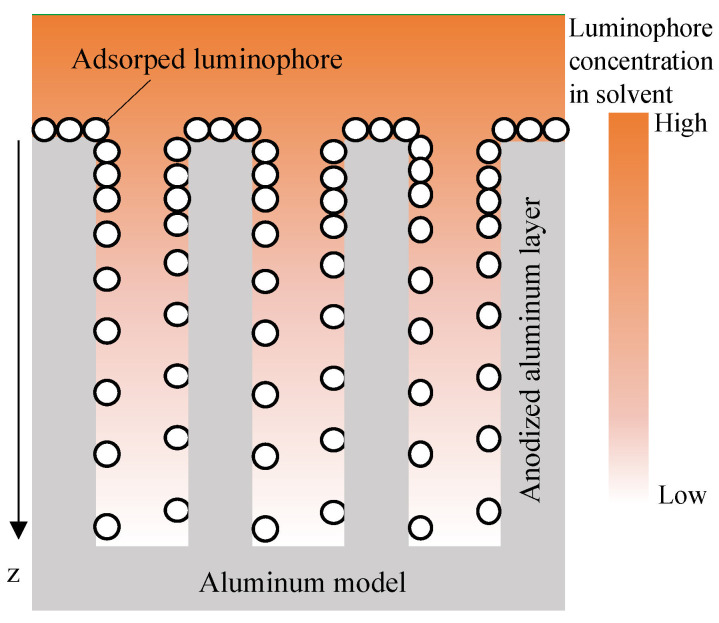
The hypothetical luminophore adsorption schematic inside the pore structure.

The pressure and temperature sensitivities of samples are summarized in Table 2. Reference pressure $P_{\mathrm{ref}}$ and temperature *T* were 100 kPa and 293 K, respectively. The thickness of the anodized aluminum layer has an effect on pressure sensitivity. The relationships between ${\mathrm{SA}}_{T10}$ to ${\mathrm{SA}}_{T30}$ and ${\mathrm{PA}}_{T20}$ to ${\mathrm{PA}}_{T60}$ clearly show that the pressure sensitivity improves as the thickness of the anodized aluminum layer increases. We consider the key of the pore structure dependence of the pressure sensitivity to be the “concentration effect” [53], which causes detrimental interactions, such as self-quenching. The concentration effects decrease the pressure sensitivity [34]. The concentration effect on the pressure and temperature sensitivities can be observed in the relationship between ${\mathrm{SA}}_{T20}$, ${\mathrm{SA}}_{C0.01}$, and ${\mathrm{PA}}_{C0.9}$. Although there is no significant difference in the temperature sensitivity, the pressure sensitivity slightly decreases as the luminophore concentration increases. The impact of concentration effects on the pressure sensitivity changes with the degree of the adsorption concentration. The degree of the adsorption concentration changes depending on the pore structures, and it is assumed to be caused by the adsorption mechanism, represented by the hypothetical adsorption schematic in Figure 12. The luminophore solution enters and fills the pore in the dipping process, and the luminophore is adsorbed on the sidewall of the pore during the solution filling process. The filling and adsorption process could take more than several seconds. Kameda et al. [23] reported that the AA-PSP with $H_{2}\mathrm{TCPP}$ at a 5 s dipping duration shows no thickness effects on the response time, and the luminophore solution was not considered to be filled in the pore well. The luminophore concentration decreases as the solution fills deeper into the pore, and the adsorption concentration is not excessively high in the deep area of the pore. On the other hand, the pore sidewall near the surface is exposed to the *fresh* solution, which keeps high luminophore concentration. It can lead to excessive adsorption and concentration effect. When the thickness of the anodized aluminum layer is thin, the area that causes the concentration effect is relatively large, resulting in lower pressure sensitivity. The difference in the pressure sensitivity between ${\mathrm{SA}}_{T10}$ to ${\mathrm{SA}}_{T30}$ and ${\mathrm{PA}}_{T20}$ to ${\mathrm{PA}}_{T60}$ could support this assumption. In addition, the concentration effect causes deeper as the pore diameter became larger because of the deeper filling of the *fresh* solution. When the pore is sufficiently deep, the area of the concentration quenching relatively decreases, and the effect of concentration effect on the pressure sensitivity is relatively low. The relationships between ${\mathrm{SA}}_{T20}$ and ${\mathrm{PA}}_{T30}$ and ${\mathrm{SA}}_{T30}$ and ${\mathrm{PA}}_{T60}$ show that the pressure sensitivity decreases when increasing the pore diameter when the thickness of the anodized aluminum layer is the approximately same.

The thicknesses of the anodized aluminum layer and the luminophore concentration have no clear effects on temperature sensitivity. On the other hand, there was a significant improvement in temperature sensitivity of ${\mathrm{PA}}_{T20}$ to ${\mathrm{PA}}_{T60}$. The temperature sensitivity of ${\mathrm{PA}}_{T20}$ to ${\mathrm{PA}}_{T60}$ is much lower than that of ${\mathrm{SA}}_{T20}$ to ${\mathrm{SA}}_{T60}$. They show a value with a magnitude smaller than one order. This suggests that the surface characteristics, such as the pore diameter of the anodized aluminum layer, deactivate the thermal quenching of $H_{2}\mathrm{TCPP}$. As mentioned in Section 4.1.1, the emission spectra of ${\mathrm{SA}}_{T20}$ and ${\mathrm{PA}}_{T30}$ are different, although the Stokes shift was the same. This can suggest that the difference in the deactivation process, such as the thermal quenching of $H_{2}\mathrm{TCPP}$ could be varied based on the anodized aluminum layer, and it caused the significant improvement of the temperature sensitivity.

#### 4.1.5. Photodegradation

**Figure 13:**
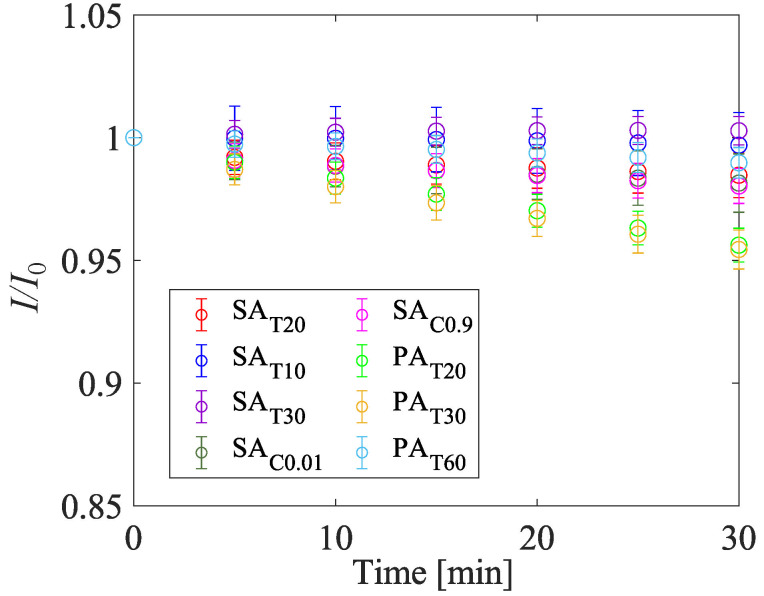
Influence of fabrication conditions on photodegradation characteristics at *P* = 100 kPa and *T* = 293 K.

The photodegradation characteristics are shown in Figure 13. Figure 13 indicates photodegradation excited by a green LED (IL-106, Hardsoft, Krakow, Poland). The photodegradation rate when an LED is used as an excitation light source, $I_{d\mathrm{LED}}$, is defined as the rate of decrease in the normalized intensity over the measurement duration as follows:

(9) $$I_{d\mathrm{LED}}=-\left(1-\frac{I_{t_{\mathrm{fin}}}}{I_{t_{0}}}\right)\frac{1}{t_{\mathrm{fin}}}\times \;100\left[\%/\min\right],$$

where $I_{t=0}$ and $I_{t=\mathrm{fin}}$ are the luminescence intensities at t=0 min and the ending time, respectively. The ending time was tfin=30 min in the present study. The photodegradation rate when the laser is used as an excitation light source, $I_{d\mathrm{Laser}}$, is defined as the rate of decrease in the normalized intensity over laser pulses as expressed in Equation (10).

(10) $$I_{d\mathrm{Laser}}=-\left(1-\frac{I_{\mathrm{fin}}}{I_{\mathrm{ini}}}\right)\frac{1}{N}\times 100\;\left[\%/\mathrm{pulse}\right]$$

Here, *N*, $I_{\mathrm{ini}}$, and $I_{N\mathrm{fin}}$ are the number of laser pulse shots, the averaged luminescence intensities of the initial $N_{\mathrm{ini}}$, and last $N_{\mathrm{fin}}$ shots, respectively, whereas $N_{\mathrm{ini}}=N_{\mathrm{fin}}=1000$. The number of total shots, *N*, was 2,000,000 in the present study. The photodegradation rate in the case with N= 1,000,000 was also calculated and the linearity of the photodegradation rate was evaluated. The photodegradation rate of the samples is summarized in Table 3 and shows no significant difference among the samples. The photodegradation rates $I_{d\mathrm{LED}}$ of all of the AA-PSP are less than 0.16%/min. The photodegradation rates of ${\mathrm{PA}}_{T20}$ and ${\mathrm{PA}}_{T30}$, which were fabricated by phosphoric acid electrolyte, are higher than those fabricated by the dilute sulfuric acid electrolyte and ${\mathrm{PA}}_{T60}$. The difference in photodegradation rate between ${\mathrm{PA}}_{T30}$ and ${\mathrm{SA}}_{T20}$ shows the effects of the diameter of the pores. The photodegradation rate increases as the diameter of the pore increases when the thickness of the anodized aluminum layer is the same.

The photodegradation rates of ${\mathrm{SA}}_{T20}$, ${\mathrm{SA}}_{C0.01}$, and ${\mathrm{SA}}_{C0.9}$ indicate that there is no significant relationship between the luminophore concentration and photodegradation rate. There is a correlation between photodegradation rate and the diameter of the pores. The photodegradation rates of ${\mathrm{SA}}_{T20}$ to ${\mathrm{PA}}_{T30}$ increase as their diameter of the pores increases. The photodegradation rate $I_{d\mathrm{Laser}}$ showed $0.93\times 10^{-5}$–$1.77\times 10^{-5}$%/pulse and $0.57\times 10^{-5}$–$1.12\times 10^{-5}$%/pulse in the cases of N= 1,000,000 and 2,000,000, respectively. The deviation of the photodegradation late $I_{d\mathrm{Laser}}$ shows the same trend as $I_{d\mathrm{LED}}$. The degradation rates are different for N= 1,000,000 and 2,000,000. This indicates that the relationship between photodegradation and time is nonlinear, and the photodegradation rate decreases as the photodegradation progresses. The photodegradation rate depends on the diameter of the pores. This trend is the same as that of the LED photodegradation case. The photodegradation during the image acquisition on the wind tunnel experiment is assumed to be approximately a few percent, and it is less than that of pyrene. This photodegradation is not neglectable but it can be applied to the long measurement time experiment, such as a conventional wind tunnel experiment.

#### 4.1.6. Solvent Resistance

**Figure 14:**
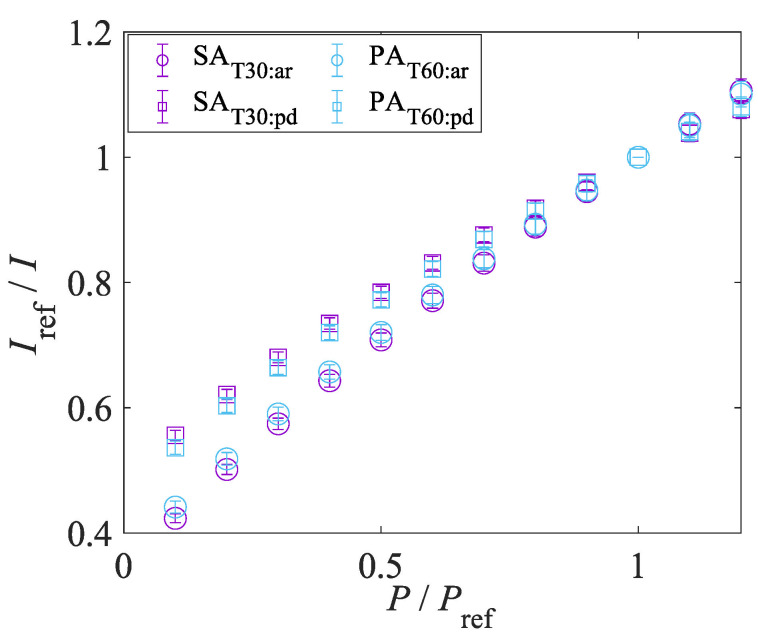
Effects of acetone rinse on Stern–Volmer-type plots of AA-PSP samples.

**Figure 15:**
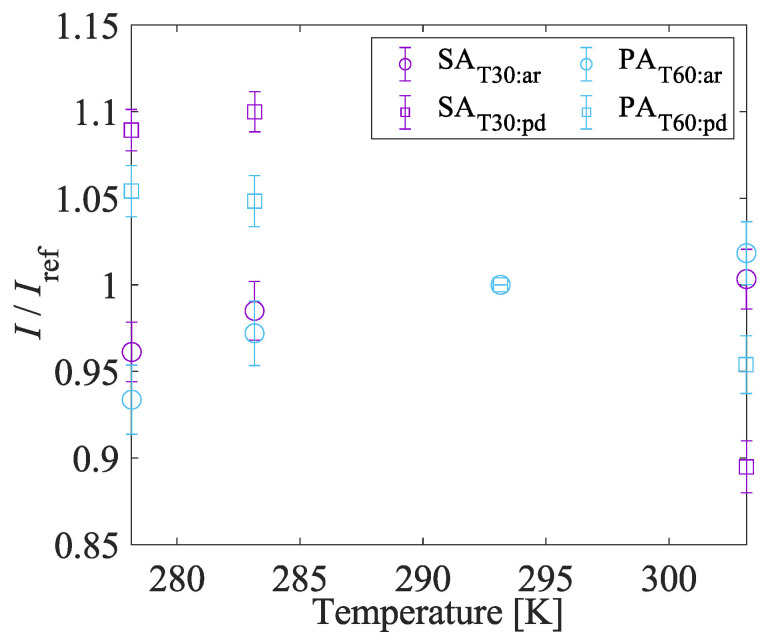
Effects of acetone rinse on the temperature sensitivity of AA-PSP samples.

The solvent resistance of the samples was investigated by rinsing with acetone which is the solvent of the luminophore. The samples after the laser photodegradation test, ${\mathrm{SA}}_{T30:\mathrm{pd}}$ and ${\mathrm{PA}}_{T60:\mathrm{pd}}$ were used in the solvent resistance test. The static calibration of the rinsed samples, ${\mathrm{SA}}_{T30\min:\mathrm{ar}}$ and ${\mathrm{PA}}_{T60:\mathrm{ar}}$, was conducted and compared with the result of the photodegraded samples ${\mathrm{SA}}_{T30:\mathrm{pd}}$ and ${\mathrm{PA}}_{T60:\mathrm{pd}}$. Figure 14 and Figure 15 show the pressure sensitivity and temperature sensitivity, respectively. Table 4 shows the comparisons of pressure sensitivity, temperature sensitivity, and signal intensity before and after the rinse process. The signal intensity of each sample was normalized by that before the rinse process. The pressure sensitivity of ${\mathrm{SA}}_{T30:\mathrm{ar}}$ and ${\mathrm{PA}}_{T60:\mathrm{ar}}$ increases 0.162%/kPa and 0.104%/kPa from ${\mathrm{SA}}_{T30:\mathrm{pd}}$ and ${\mathrm{PA}}_{T60:\mathrm{pd}}$, respectively. The temperature sensitivity of ${\mathrm{SA}}_{T30:\mathrm{ar}}$ and ${\mathrm{PA}}_{T60:\mathrm{ar}}$ decrease $6.97\times 10^{-2}$% and 0.714% from ${\mathrm{SA}}_{T30:\mathrm{pd}}$ and ${\mathrm{PA}}_{T60:\mathrm{pd}}$, respectively. These changes in pressure and temperature sensitivity are favorable for a PSP. On the other hand, the signal intensity of both cases significantly decreases by approximately 20% after the rinse process. The luminophore $H_{2}\mathrm{TCPP}$ is adsorbed onto the anodized aluminum layer by chemical bonding and $H_{2}\mathrm{TCPP}$ cannot be removed with any polar solvents [23]. Therefore, the samples fabricated in the present study after the rinse process still worked as a PSP. The mechanism of increase in the pressure sensitivity and decrease in the signal intensity is not revealed. These characteristics suggest that the performance of the proposed PSP could be recovered to some extent by washing with polar solvents, even if the AA-PSP was polluted. This solvent resistance is important for the practical PSP.

### 4.2. Dynamic Characteristics

#### 4.2.1. Resonance Tube

**Figure 16:**
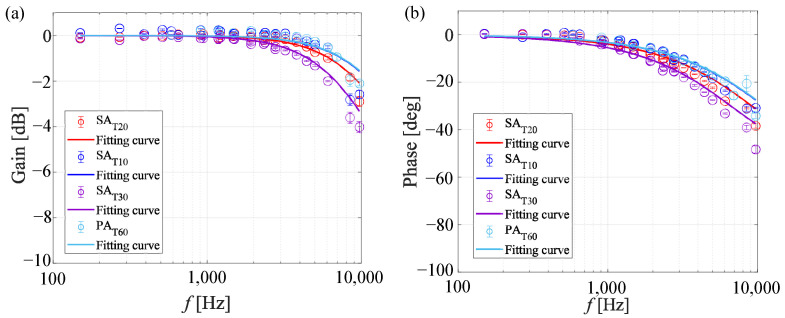
Bode plots of the AA-PSP at atmospheric pressure and $T_{ref}=293\left[K\right]$: (**a**) gain and (**b**) phase with fitting curves.

Figure 16 shows Bode plots for ${\mathrm{SA}}_{T10}$, ${\mathrm{SA}}_{T20}$, ${\mathrm{SA}}_{T30}$, and ${\mathrm{PA}}_{T60}$. The solid line is the frequency response predicted using the parameters fitted with the experimental data. The gain and phase delays were increased and improved as the thickness of the anodized aluminum layer decreased. The estimated cut-off frequency is approximated by extrapolation based on the first-order transfer function. The estimated cut-off frequencies were higher than 10 kHz, except for ${\mathrm{SA}}_{T30}$. The difference in the estimated cut-off frequencies of ${\mathrm{SA}}_{T10}$, ${\mathrm{SA}}_{T20}$, and ${\mathrm{SA}}_{T30}$ shows the effects of the thickness of the anodized aluminum layer. The estimated cut-off frequency increases as the thickness of the anodized aluminum layer decreases. This trend agrees well with that predicted by the theory expressed in Equation (3). The cut-off frequencies of ${\mathrm{SA}}_{T10}$ and ${\mathrm{PA}}_{T60}$ are approximately 15 kHz and are almost the same as each other.

The layer thicknesses of ${\mathrm{SA}}_{T30}$ and ${\mathrm{PA}}_{T60}$ were approximately the same, and thus the difference in the frequency response between them is considered to be mainly caused by the difference in the pore diameter. The estimated cut-off frequency increases as the pore diameter increases. The diffusivity coefficients identified for each AA-PSP by fitting the two-layer model proposed by Nonomura and Asai [49] are shown in Table 5. The bottom layer and hiding factor of the AA-PSPs were assumed to be zero because the AA-PSP consists of a single layer and the luminophore is adsorbed on the binder. Kasai et al. [24] estimated the diffusivity coefficient using this two-layer model and gradient descent-based optimization and reported that the diffusivity coefficients of the PSPs did not change significantly with the ambient pressure. The ambient pressure of the present research was atmospheric pressure. The sample ${\mathrm{PA}}_{T60}$ has the highest diffusivity coefficient in this case. The diffusion coefficient of ${\mathrm{SA}}_{T30}$ is almost the same as the estimated value in previous research [24]. The comparison of the diffusion coefficient of ${\mathrm{SA}}_{T30}$ and ${\mathrm{PA}}_{T60}$ shows that the diffusion coefficient increases as the pore diameter increases. The trend agrees well with the theory expressed in Equation (4).

#### 4.2.2. Shock Tube Experiment

**Figure 17:**
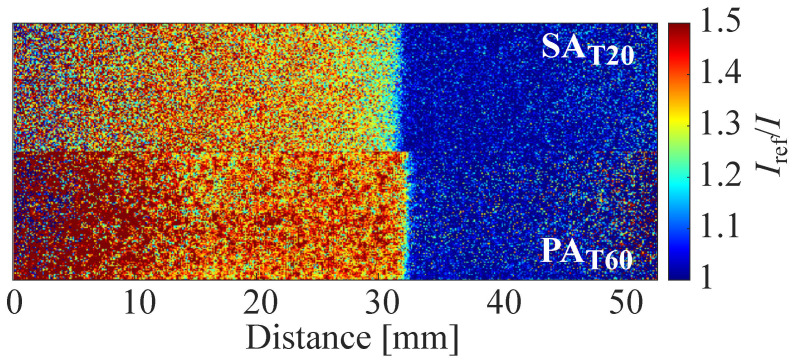
Shock wave visualization two kinds of AA-PSPs.

Figure 17 shows the visualized normal shock wave by ${\mathrm{SA}}_{T20}$ and ${\mathrm{PA}}_{T60}$. These two types of samples were employed in the shock tube experiment because of their high cut-off frequency, which is over 10 kHz, signal intensity, and pressure sensitivity. The shock waves are observed without strong blurring in both cases, but the edge of the normal shock wave visualized by ${\mathrm{PA}}_{T60}$ is clearer than that of ${\mathrm{SA}}_{T20}$.

The time constants of the AA-PSP sample to the normal shock wave were evaluated for more quantitative discussion. The analysis procedures for evaluation of the time constant are based on the methods of Numata et al. [38] and Yomo et al. [39]. There are four steps in this procedure. The first step is the conversion from distance to the time after the shock wave passed. The second step is spatial averaging of the signal intensity ratio in the direction orthogonal to the shock propagation. For the third step, the fitting curves are calculated based on the first-order model, as shown in Equation (11)

(11) $$f\left(t\right)=1-e^{-\frac{t}{\tau }}.$$

Note that the fitting was conducted with the first-order model, which is simpler than the model employed for the frequency response characterization. Sakaue et al. [54] reported that the response of the PSP has slow and fast components, but those two different time scale responses could not be explained by the first-order model [55]. Finally, consideration of the effect of the exposure time is necessary when the time constant τ in Equation (11) is estimated from the measured data by fitting the curve. A convolution integral of the pure response curve and the square pulse function was calculated. The detail of this method is described by Yomo et al. [39]. The time constant and the time until the signal intensity ratio reaches 90% are defined as the time constant and the rise time, respectively. The 90% rise time in Equation (12) is often used for the evaluation of the time response of PSPs.

(12) $$\tau_{90\%}=\tau \ln10$$

**Figure 18:**
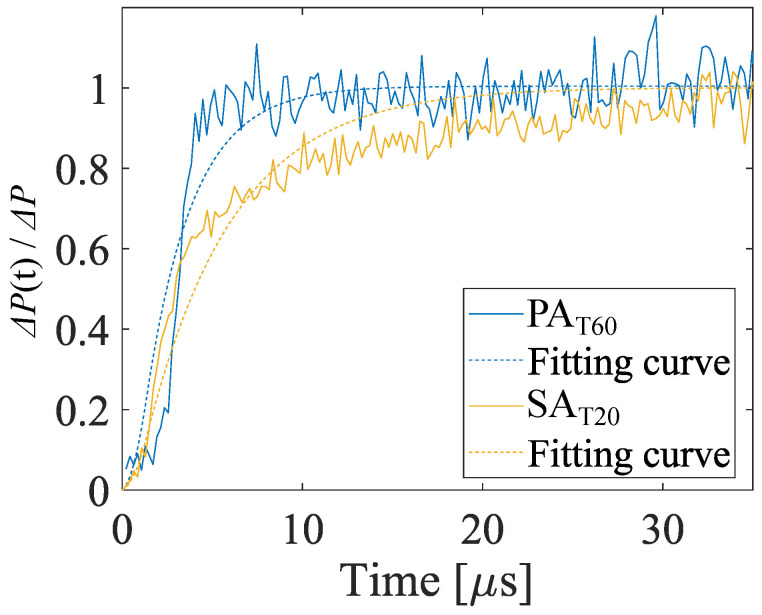
The response of two kinds of AA-PSPs to a step pressure rise.

Figure 18 shows the relation between the time and pressure ratios. The solid and dashed lines indicate the measured data and the fitting curves with compensation of the exposure time. The time constant and the rise time are evaluated based on the fitting curves. As a result, the time constants of ${\mathrm{SA}}_{T10}$ and ${\mathrm{PA}}_{T60}$ were 4.87 µs and 2.60 µs, respectively, and the rise times were 11.2 µs and 5.99 µs, respectively. The time constants and rise times are summarized in Table 6. These values are equivalent to 33 kHz and 61 kHz based on the cut-off frequency of the first-order model. The results mean these AA-PSPs have sufficient responsiveness for the measurement of the phenomena of the order of 10 kHz.

## 5. Conclusions

In the present study, we developed and evaluated an anodized-aluminum pressure-sensitive paint (AA-PSP) with new formulations of free-base porphyrin, $H_{2}\mathrm{TCPP}$, as an optical unsteady pressure sensor. The effects of the thickness of the anodized aluminum layer, the diameter of the pore, and the concentration of the luminophore were investigated.

The excitation spectra of the reference plate show two high-intensity spectra, which are around 425 nm and also longer than 520 nm. There is a difference in excitation spectra between the reference plate and ${\mathrm{PA}}_{T60}$. The luminescence lifetime of ${\mathrm{SA}}_{T20}$ at the atmospheric condition was 2.32 ns, and it is sufficiently shorter than the diffusion time. These characteristics suggest that a high-repeatable, high-power laser, such as a high Nd:YAG laser and a Nd:YLF laser, are valid as an excitation light source of the AA-PSP. The combination with the AA-PSP and a high-repeatable, high-power laser can measure a pressure distribution of high-frequency oscillation phenomena at a high sampling rate.

In the present study, the AA-PSP samples were fabricated with different preparation conditions. The pressure sensitivities of the samples were in the range of 0.33–0.54%/kPa and their temperature sensitivities were in the range of 0.07–1.46%/K. The pressure sensitivity shows the dependency of the pore structure, and the mechanism of this dependency was assumed based on a hypothetical adsorption mechanism. The pressure and temperature sensitivities of these AA-PSPs are sufficiently high for the measurement of the high-frequency phenomena in supersonic flowfields. The samples showed resistance against photodegradation, and it illustrates that $H_{2}\mathrm{TCPP}$ is better than pyrene as a luminophore for a fast PSP for the conventional wind tunnel experiment from the aspect of the photodegradation. The pressure and temperature sensitivities were shown to be improved after the acetone rinse process while the signal intensity decreased.

The cut-off frequencies were higher than 10 kHz except for ${\mathrm{SA}}_{T30}$. The cut-off frequencies of ${\mathrm{SA}}_{T10}$ and ${\mathrm{PA}}_{T60}$ are almost the same and approximately 15 kHz. The time constant to the normal shock wave of ${\mathrm{SA}}_{T10}$ and ${\mathrm{PA}}_{T60}$ were 4.87 µs and 2.60 µs. These values are equivalent to 33 kHz and 61 kHz based on the corner frequency of the first-order model. These results show the cut-off frequency of the new AA-PSP is sufficiently high for the measurement of 10 kHz order phenomena. The fast responsiveness, the sufficiently high pressure sensitivity, and the resistance to photodegradation are preferable characteristics of a practical fast PSP.

## Figures and Tables

**Table 1:** Fabrication conditions.

Sample Name	${\mathrm{SA}}_{\mathrm{Ru}}$	${\mathrm{SA}}_{T10}$	${\mathrm{SA}}_{T20}$	${\mathrm{SA}}_{T30}$	${\mathrm{SA}}_{C0.01}$	${\mathrm{SA}}_{C0.9}$	${\mathrm{PA}}_{T20}$	${\mathrm{PA}}_{T30}$	${\mathrm{PA}}_{T60}$
Electrolyte	Sulfuric acid						Phosphoric acid		
Pores diameter (nm)	20–100						165		
Anodization time (min)	20	10	20	30	20	20	20	30	60
Layer thickness (µm)	4.9	1.7	4.9	8.5	4.9	4.9	4	4.9	9.8
Luminophore	Ru(dpp)_3_	$H_{2}\mathrm{TCPP}$							
Luminophore concentration (mM *)	0.1				0.01	0.9	0.1		

* M: molar concentration (mol/L).

**Table 2:** Summary of pressure sensitivity and temperature sensitivity.

Sample	${\mathrm{SA}}_{T10}$	${\mathrm{SA}}_{T20}$	${\mathrm{SA}}_{T30}$	${\mathrm{SA}}_{C0.01}$	${\mathrm{SA}}_{C0.9}$	${\mathrm{PA}}_{T20}$	${\mathrm{PA}}_{T30}$	${\mathrm{PA}}_{T60}$
$S_{P}$	0.437	0.499	0.544	0.505	0.485	0.331	0.377	0.505
(%/kPa)								
$S_{T}$	1.461	0.526	0.815	0.726	0.774	0.104	0.072	0.091
(%/K)								

**Table 3:** Summary of photodegradation characteristics at *P* = 100 kPa and *T* = 293 K.

Sample	${\mathrm{SA}}_{T10}$	${\mathrm{SA}}_{T20}$	${\mathrm{SA}}_{T30}$	${\mathrm{SA}}_{C0.01}$	${\mathrm{SA}}_{C0.9}$	${\mathrm{PA}}_{T20}$	${\mathrm{PA}}_{T30}$	${\mathrm{PA}}_{T60}$
$I_{d\mathrm{LED}}$	0.010	0.051	−0.009	0.062	0.066	0.146	0.152	0.034
(%/min)								
$I_{d\mathrm{Laser}}$								
$(\times 10^{-5}\%/\mathrm{pulse})$								
N= 1,000,000	0.93	1.07	1.31	1.26	1.28	1.59	1.77	1.29
N= 2,000,000	0.57	0.63	0.87	0.78	0.77	1.04	1.12	0.81

**Table 4:** Effects of acetone rinse on photodegraded samples.

Sample No.	${\mathrm{SA}}_{T30:\mathrm{pd}}$	${\mathrm{SA}}_{T30:\mathrm{ar}}$	${\mathrm{PA}}_{T60:\mathrm{pd}}$	${\mathrm{PA}}_{T60:\mathrm{ar}}$
$S_{P}$	0.482	0.644	0.520	0.624
(%/kPa)				
$S_{T}$	9.32 × $10^{-2}$	2.35 × $10^{-2}$	0.839	0.125
(%/K)				
Signal intensity	1	0.81	1	0.80

**Table 5:** Estimated cut-off frequencies and diffusion coefficients of AA-PSPs.

Sample	${\mathrm{SA}}_{T10}$	${\mathrm{SA}}_{T20}$	${\mathrm{SA}}_{T30}$	${\mathrm{PA}}_{T60}$
Estimated cut-off frequency (kHz)	15.0	12.5	9.0	15.2
Diffusion coefficient (m${}^{2}$/s)	$1.05\times 10^{-7}$	$7.25\times 10^{-7}$	$1.58\times 10^{-6}$	$3.53\times 10^{-6}$
Theoretical diffusion coefficient (m${}^{2}$/s)	$2.40\times 10^{-7}$	$2.40\times 10^{-7}$	$2.40\times 10^{-7}$	$7.51\times 10^{-7}$

**Table 6:** Shock tube experiment condition and results.

Sample	Mach Number of Normal Shock Wave	The Time Constant (µs)	The 90% Rise Time (µs)
${\mathrm{SA}}_{T20}$	1.41	4.87	11.2
${\mathrm{PA}}_{T60}$	1.63	2.60	5.99

## Data Availability

Data sharing is not applicable.
